# Silicon in Poultry Nutrition: Biological Roles, Bioavailability, and Implications for Skeletal Health and Performance

**DOI:** 10.1007/s12011-026-05053-1

**Published:** 2026-03-11

**Authors:** M. Naeem

**Affiliations:** Department of Poultry Science, Auburn University, Auburn, AL 36849 USA

**Keywords:** bioavailability, bone mineralization, broilers, eggshell quality, laying hens, silicon

## Abstract

Silicon has traditionally received limited attention in poultry nutrition compared to macro and trace minerals, yet increasing evidence suggests it plays important physiological roles relevant to modern intensive production systems. This review compiles current knowledge on the presence, metabolism, and nutritional value of silicon in poultry, focusing on skeletal health, performance results, and egg quality. Silicon is common in plant and mineral feed ingredients but exists in various chemical forms that vary widely in solubility and biological availability. Although not traditionally considered essential, silicon appears to support collagen production, cartilage growth, and the mineralization of bone and connective tissue. Reported benefits of dietary supplementation include stronger tibias in broilers, improved bone stability in laying hens, and, in some cases, better eggshell quality and overall performance. Potential effects on gut health and immune function have also been suggested. However, results across studies are inconsistent. Variations likely stem from differences in silicon sources’ chemical structure, interactions with dietary calcium and phosphorus, environmental stressors, and the bird’s developmental stage. Bioavailable forms like orthosilicic acid seem more effective than less soluble silicates, but standardized methods for measuring availability are limited. Importantly, the gap between known requirements and typical dietary intake remains unclear, and silicon is not yet universally recognized as essential for poultry. Overall, silicon is a promising yet incompletely understood nutrient with practical potential to reduce skeletal issues, enhance longevity in layers, and improve welfare. Future research should focus on elucidating absorption pathways, establishing safe and effective supplementation levels, and exploring interactions with key minerals and gut health. A clearer understanding of silicon biology will help incorporate this overlooked element into evidence-based poultry nutrition strategies.

## Introduction and Historical Perspective on Silicon

The role of minerals in poultry nutrition has traditionally focused on elements such as calcium, phosphorus, sodium, zinc, iron, and manganese, owing to their clearly established requirements and deficiency syndromes. For many decades, silicon occupied a marginal and often controversial position within this framework. Although silicon is one of the most abundant elements in the Earth’s crust and is widely present in plant-derived feedstuffs, its nutritional relevance for animals, particularly poultry, was long underestimated or dismissed as incidental [1]. Early nutritional paradigms regarded silicon largely as an inert component of structural materials or as an unavoidable contaminant of feed rather than as a biologically active trace element [2, 3]. However, progressive experimental evidence accumulated over the past five decades has challenged this perception and repositioned silicon as a potentially essential micronutrient with specific physiological functions in poultry.

The historical journey of silicon in poultry nutrition is closely associated with foundational research in the latter half of the twentieth century, particularly the pioneering work of Carlisle [4]. In a landmark study, Carlisle [4] demonstrated for the first time that silicon is essential for normal growth and skeletal development in chicks. Using a purified amino acid-based diet deliberately formulated to be low in silicon, Carlisle [4] observed that day-old cockerels developed severe growth retardation within two to three weeks, accompanied by skeletal abnormalities and organ atrophy. In contrast, chicks receiving the same diet supplemented with silicon exhibited near-normal growth rates and structural development. This study was seminal because it provided controlled experimental evidence that silicon deficiency alone, independent of other nutrients, could impair growth and development in poultry.

Building on this foundational work, Carlisle [5] further explored the biological role of silicon and proposed its classification as an essential trace element in animal nutrition. This concept represented a major shift in thinking, as essentiality traditionally required a clear demonstration of deficiency symptoms, physiological specificity, and restoration of function upon repletion. Through a series of biochemical and histological analyses, Carlisle [5] reported that silicon deficiency in chicks resulted in pronounced abnormalities in bone and cartilage, primarily due to disruptions in the formation of the organic matrix rather than defects in mineral deposition per se. These findings suggested that silicon plays a fundamental role in connective tissue metabolism, particularly in the synthesis and stabilization of collagen and glycosaminoglycans, which form the scaffold for subsequent mineralization.

The importance of silicon for skeletal integrity was reinforced by Carlisle [6], who demonstrated that silicon is required for normal skull formation in chicks independently of vitamin D status. In this study, silicon-deficient chicks displayed gross deformities in skull architecture, including narrowing of the frontal bones and depression at the parietal junction, despite adequate vitamin D supply. Biochemical analysis revealed significantly reduced collagen content in the bones of silicon-deficient birds, confirming that silicon deficiency compromises the organic bone matrix rather than mineral accretion alone. This work was particularly influential because it disentangled the effects of silicon from those of vitamin D and calcium, two nutrients traditionally considered central to skeletal health in poultry.

Despite these compelling findings, the broader poultry nutrition community remained cautious in embracing silicon as an essential nutrient. One reason was that practical poultry diets based on cereals, oilseed meals, and forages typically contain relatively high concentrations of silicon derived from plant cell walls and soil contamination [7–9]. As a result, overt deficiency symptoms were unlikely to manifest under commercial conditions, leading to the assumption that dietary silicon was always sufficient. Additionally, early analytical methods for silicon quantification were limited, and the bioavailability of different silicon forms in feed ingredients was poorly understood [10]. These challenges contributed to a prolonged period during which silicon received limited attention in poultry feeding standards and nutrient requirement tables.

Modern broiler strains, in particular, exhibit accelerated growth rates that often outpace skeletal development, leading to leg disorders, fractures, and compromised welfare [11, 12], leading to lameness, restricting the ability to get access to feed and water [13]. In this context, researchers revisited the potential role of silicon in supporting bone strength and the development of connective tissue [10]. Studies such as those by Elliott and Edwards [14] evaluated dietary silicon supplementation in broilers and reported mixed results, with no consistent improvements in growth or tibia ash at supplemental silicon inclusion levels typically ranging from approximately 100 to 500 mg/kg of diet, depending on source and solubility. These findings contributed to ongoing debate regarding the practical significance of silicon supplementation, highlighting the complexity of its biological role and the influence of dosage, form, and developmental stage.

Advances in analytical techniques and experimental design in the early twenty-first century enabled more nuanced investigations into silicon metabolism and function. Carlisle [2] synthesized decades of research, reaffirming silicon’s role as an essential trace element involved in connective tissue formation, and emphasized its high concentration in osteogenic cells and mitochondria. This work highlights the concept that silicon plays a crucial role in the early stages of bone formation by facilitating collagen synthesis and matrix organization, rather than serving as a direct mineral component of hydroxyapatite. Such insights helped reconcile earlier inconsistencies in performance studies by suggesting that silicon’s effects may be most pronounced during early growth phases or under conditions of marginal mineral supply.

Concurrently, research began to explore novel sources and forms of dietary silicon with improved bioavailability [10]. Traditional silicon sources in poultry diets, such as sand, silica grit, and plant-derived silicates, are largely insoluble and poorly absorbed [15]. This realization prompted investigations into soluble and nanoscale silicon compounds that could enhance biological uptake. For example, Sgavioli et al. [16] evaluated the inclusion of silicon in broiler drinking water and reported significant increases in tibial mineral content, including phosphorus, zinc, copper, and manganese, without adverse effects on growth performance. These findings suggested that bioavailable silicon can positively influence mineral metabolism and bone composition even when growth responses are subtle or absent.

Further reinforcing this perspective, Burton et al. [17] examined the efficacy and stability of a novel silica supplement in broilers and highlighted that the adequacy of bioavailable silicon in poultry diets had not been systematically considered for several decades. Their work demonstrated that specific silicon formulations could enhance bone development, prompting renewed interest in revisiting silicon requirements under modern production conditions. Similarly, in the earlier study, Scholey et al. [15] reported that a novel pH-neutral silicon supplement exhibited superior bioavailability compared with conventional sources and significantly improved tibia breaking strength in young broilers, highlighting the importance of form and solubility in determining biological responses.

Parallel research efforts in Eastern Europe and Asia contributed additional evidence supporting the nutritional relevance of silicon. Mustafina and Rakhmatullin [18] reported that ultrafine silicon dioxide particles improved nutrient digestibility, energy utilization, and live weight gain in broilers, suggesting that silicon may exert indirect effects on performance through modulation of digestive processes. Nikulin et al. [19] further demonstrated that inclusion of ultrafine silicon dioxide optimized protein composition in broiler meat, enhancing its nutritional and organoleptic properties. Although these studies often emphasized performance outcomes rather than classical deficiency symptoms, they expanded the conceptual scope of silicon nutrition beyond skeletal health alone.

Importantly, not all studies have reported positive effects of silicon supplementation, and this variability has shaped the historical narrative of silicon in poultry nutrition. Pritchard et al. [20] found that silicon supplementation altered serum mineral profiles in male broilers but did not improve bone density or strength, suggesting that silicon’s benefits may depend on factors such as growth rate, mechanical loading, baseline mineral status, and developmental timing. These findings underscore that silicon is not a simple growth-promoting additive but a nutrient whose physiological role is context-dependent and intricately linked to other aspects of mineral metabolism.

From a historical perspective, the evolving understanding of silicon in poultry nutrition mirrors broader trends in animal nutrition science. Initial discovery of essentiality was followed by skepticism due to practical feeding conditions and inconsistent performance responses. Subsequent advances in analytical chemistry, molecular biology, and materials science enabled researchers to revisit earlier assumptions and explore new forms of supplementation. Today, silicon is increasingly recognized as a biologically active micronutrient with specific roles in connective tissue development, skeletal integrity, and mineral interactions, even if its precise dietary requirement remains undefined [3]. In summary, the historical trajectory of silicon research in poultry nutrition reflects a gradual shift from neglect to cautious recognition. Early experimental work firmly established silicon’s essentiality for normal growth and skeletal development in chicks. Subsequent studies expanded the understanding of its biochemical functions, particularly in collagen synthesis and bone matrix formation. Although commercial poultry diets typically supply ample silicon, modern production challenges and the availability of bioavailable silicon sources have renewed interest in its nutritional value. This historical foundation provides the necessary context for a deeper examination of silicon chemistry, metabolism, and practical applications in poultry feeding.

The objective of this review is to critically synthesize current knowledge on the nutritional significance of silicon in poultry, with emphasis on its biological roles, chemical forms, and bioavailability. Specifically, the review aims to evaluate the involvement of silicon in skeletal development, mineral interactions, gut health, and nutrient utilization, and to assess its indirect effects on growth performance, carcass traits, egg quality, and reproductive efficiency. This review focuses on silicon primarily in its nutritional context, particularly bioavailable forms relevant to physiological functions, rather than on silicon-containing compounds used as functional feed additives for purposes such as mycotoxin binding, ammonia adsorption, litter management, or feed processing. In addition, this review seeks to identify limitations in existing research and highlight future directions for optimizing the practical application of silicon in modern poultry nutrition.

## Chemical Forms, Sources, and Bioavailability of Silicon

Understanding the nutritional value of silicon for poultry requires careful consideration of its chemical forms, dietary sources, and bioavailability. Unlike many trace minerals that exist in relatively uniform ionic forms within feeds, silicon occurs in a wide variety of chemical structures that differ markedly in solubility, reactivity, and physiological availability [8, 21–24]. This diversity has been a central reason for historical inconsistencies in experimental outcomes and for the prolonged debate regarding silicon’s nutritional significance in poultry. The biological effects of silicon are not determined solely by its total concentration in the diet, but rather by the fraction that is soluble, absorbable, and metabolically active [8].

In natural feed ingredients, silicon is primarily present as silicon dioxide or as complex silicates integrated into plant cell walls [25]. Cereals, oilseed meals, and forage-based ingredients contain substantial quantities of silicon, often ranging from 0.1 to 10% of dry matter, depending on plant species and growing conditions [26]. Although total silicon concentrations in basal feed ingredients may range widely (approximately 0.1–10% of dry matter), the majority of this silicon exists in insoluble plant- or soil-derived forms with low biological availability. Reported supplementation levels, therefore, refer specifically to added, bioavailable silicon sources rather than total dietary silicon content. Grass and cereal hulls, in particular, accumulate high levels of silicon in the form of phytoliths, which are rigid silica structures deposited within plant tissues [27]. Although these forms contribute significantly to total dietary silicon, they are largely insoluble under gastrointestinal conditions and therefore exhibit very low bioavailability for poultry. Carlisle [5] emphasized that the abundance of silicon in plant-based feeds should not be equated with nutritional adequacy, as only a small proportion exists in forms that can be absorbed and utilized by animal tissues.

Silicon in feed ingredients may also originate from soil contamination during harvesting and processing. Sand, dust, and grit contribute additional silicon dioxide, further inflating analytical values of total silicon content [28]. Historically, this led to the assumption that poultry diets were inherently rich in silicon and incapable of inducing deficiency. However, studies using purified diets have clearly demonstrated that total silicon content is a poor predictor of biological availability. Carlisle [4] showed that chicks fed amino acid-based diets containing negligible amounts of soluble silicon developed severe deficiency symptoms, despite the presence of trace inorganic contaminants. This distinction between total silicon and bioavailable silicon remains fundamental to interpreting nutritional studies and designing supplementation strategies.

From a chemical standpoint, silicon exists in both inorganic and organic forms, with markedly different physiological implications [8, 29]. Inorganic silicon is most commonly encountered as silicon dioxide, either in crystalline or amorphous forms, and as various silicate salts [30]. Crystalline silica, such as quartz, is essentially inert and insoluble, rendering it biologically unavailable [31]. Amorphous silica, while more reactive than crystalline forms, generally exhibits limited solubility under physiologically relevant conditions. Although pH varies along the avian gastrointestinal tract, including acidic conditions in the proventriculus and gizzard that may promote partial dissolution, the overall solubility and biological availability of amorphous silica remain low compared with soluble forms such as orthosilicic acid [32]. Traditional feed additives such as silica grit and sand fall into this category and have generally shown minimal nutritional value for poultry. Majewska et al. [33] reported that silica grit had adverse or neutral effects on turkey performance, further illustrating the limited usefulness of insoluble silicon sources in poultry nutrition.

In contrast, soluble forms of silicon, particularly orthosilicic acid and its stabilized derivatives, are considered the primary biologically active species [34]. Orthosilicic acid is formed when silicon dioxide dissolves in water at low concentrations, yielding monomeric silicic acid molecules that can be readily absorbed across the intestinal epithelium [22]. Carlisle [6] proposed that orthosilicic acid is the physiologically relevant form of silicon involved in connective tissue metabolism and bone formation. However, orthosilicic acid is inherently unstable and prone to polymerization, which limits its natural availability in feeds and complicates its use as a dietary supplement.

To overcome these limitations, researchers have explored stabilized and modified silicon compounds designed to enhance solubility and prevent polymerization. Scholey et al. [15] evaluated a novel pH-neutral silicon supplement and demonstrated substantially higher in vitro and in vivo bioavailability compared with conventional silicon sources. Their work showed increased silicon concentrations in blood plasma and bone tissue of broiler chicks, accompanied by improvements in tibia ash and breaking strength. These findings highlighted that chemical stabilization is a critical determinant of silicon bioavailability and biological efficacy.

Another important category of silicon sources includes ultrafine and nanoscale silicon dioxide particles [35]. Advances in materials science have enabled the production of silicon dioxide with extremely small particle sizes, greatly increasing surface area and reactivity [36]. Mustafina and Rakhmatullin [18] investigated ultrafine silicon dioxide in broiler diets and reported improvements in nutrient digestibility, energy utilization, and live weight gain. Although silicon dioxide itself is traditionally considered poorly soluble, reduction to ultrafine particle size appears to enhance its interaction with the gastrointestinal environment, potentially increasing the formation of absorbable silicic acid. Nikulin et al. [19] further demonstrated that ultrafine silicon dioxide optimized protein composition in broiler meat, suggesting systemic metabolic effects beyond the digestive tract.

Silicon can also be delivered through drinking water, an approach that bypasses some of the limitations associated with feed-based supplementation. Sgavioli et al. [16] supplemented broiler drinking water with soluble silicon and observed significant increases in tibial mineral content without adverse effects on growth performance. This method ensures direct exposure of the intestinal mucosa to soluble silicon species and may be particularly effective during early growth phases when skeletal development is most rapid. Water-based supplementation also allows precise control of dosage and reduces interactions with feed components that may bind or precipitate silicon.

Organic silicon compounds represent another promising avenue for improving bioavailability. These compounds typically involve silicon bonded to carbon-containing groups, which can enhance stability and absorption. Although much of the research on organic silicon has been conducted in non-poultry species, findings are relevant for understanding potential mechanisms in birds. Carlisle [2] noted that organically bound silicon exhibits higher retention in connective tissues compared with inorganic forms, supporting its role in collagen synthesis and matrix organization. In poultry-specific contexts, silicate-based complex minerals and arginine silicate inositol complexes have shown beneficial effects on bone metabolism and egg quality, indirectly implicating improved silicon availability [37].

Despite these advances, silicon bioavailability in poultry remains influenced by numerous dietary and physiological factors. Interactions with other minerals, particularly calcium, phosphorus, and magnesium, can modulate silicon absorption and utilization. Kayongo-Male and Julson [38], working with turkeys fed semi-purified diets, reported antagonistic effects between high dietary silicon and calcium and magnesium metabolism, indicating that excessive silicon intake under controlled dietary conditions may impair mineral balance. These findings underscore that bioavailability is not solely a function of chemical form, but also of dietary context and inclusion level.

The gastrointestinal environment of poultry further shapes silicon bioavailability. The pH gradients, digestive enzymes, and microbial activity can influence the dissolution and transformation of silicon compounds. Ruttanavut et al. [39] demonstrated that dietary silicic acid powder enhanced intestinal villus height and epithelial cell proliferation in chickens, suggesting that silicon may modify gut morphology in ways that indirectly affect nutrient absorption. Such effects could increase the efficiency of silicon uptake itself, creating a positive feedback loop between gut health and mineral utilization.

Importantly, age and physiological stage appear to influence silicon absorption efficiency. Available evidence suggests young chicks exhibit higher rates of silicon uptake and retention in bone tissue compared with older birds, consistent with the element’s role in early skeletal development. Scholey et al. [15] observed more pronounced increases in bone ash and strength in young broilers receiving bioavailable silicon supplements, whereas effects diminished with age. This developmental sensitivity aligns with early findings [4] and suggests that silicon supplementation strategies should be tailored to specific production phases.

In summary, the nutritional value of silicon for poultry is inseparable from its chemical form, dietary source, and bioavailability. While total silicon content in conventional poultry diets is often high, much of it exists in insoluble forms with minimal biological relevance. Soluble and stabilized forms of silicon, including orthosilicic acid derivatives, ultrafine silicon dioxide, and organic silicon complexes, exhibit markedly higher bioavailability and have demonstrated positive effects on bone development, mineral metabolism, and nutrient utilization. However, silicon bioavailability is modulated by interactions with other dietary minerals, gastrointestinal conditions, and the physiological stage of the bird. Recognizing these complexities is essential for interpreting experimental findings and for designing effective silicon supplementation strategies.

## Silicon Metabolism, Absorption, and Physiological Roles

The metabolic fate of silicon in poultry is a critical determinant of its nutritional value and biological significance. Unlike many essential minerals whose absorption, transport, and storage pathways are well defined, silicon metabolism in poultry is comparatively less understood and has historically been regarded as passive or nonspecific. However, accumulating experimental evidence indicates that silicon is actively involved in several physiological processes, particularly those related to connective tissue development, skeletal integrity, and mineral homeostasis. Understanding how silicon is absorbed, distributed, and utilized within the avian body provides essential context for interpreting its functional roles and for optimizing dietary strategies.

Orthosilicic acid is widely regarded as the physiologically relevant absorbable form of silicon in mammals; however, the specific mechanisms governing silicon absorption in avian species are not yet fully characterized. Available avian studies suggest that soluble forms of silicon are more readily absorbed than insoluble forms, but the extent to which orthosilicic acid is absorbed via passive diffusion in poultry remains uncertain and is largely inferred from mammalian models. When present in the diet or drinking water, orthosilicic acid can pass through the intestinal epithelium by passive diffusion, driven by concentration gradients. Carlisle [6] proposed that silicon absorption does not rely on a highly specific transport system, but rather on its presence in a monomeric, soluble state. This explains why insoluble forms such as crystalline silica and sand contribute little to silicon status despite their abundance in many poultry diets.

The efficiency of silicon absorption in poultry is influenced by both dietary and physiological factors. Solubility is paramount, as polymerized or particulate forms of silicon are poorly absorbed. Scholey et al. [15] demonstrated that a stabilized, pH-neutral silicon supplement resulted in significantly higher blood silicon concentrations compared with conventional silicon sources, confirming that chemical form directly affects absorption efficiency. Similarly, Sgavioli et al. [16] reported increased silicon deposition in bone tissue when soluble silicon was supplied through drinking water, highlighting the importance of the delivery route in determining bioavailability.

Once absorbed, silicon is distributed throughout the body, with preferential accumulation in connective tissues, bone, skin, and cartilage [8]. Carlisle [5] reported high concentrations of silicon in osteogenic cells and their mitochondria, suggesting an active metabolic role rather than passive deposition. This localization is consistent with silicon’s proposed function in early stages of bone formation, particularly in the synthesis and stabilization of the organic matrix. In poultry, rapidly developing skeletal tissues represent major sinks for absorbed silicon, especially during early growth phases when collagen production and matrix organization are most intense [16].

Reported plasma silicon concentrations in poultry are generally low; however, baseline physiological levels and regulatory mechanisms have not been comprehensively defined in avian species [40]. Scholey et al. [15] observed dose-dependent increases in blood silicon following supplementation, but these increases were modest compared with tissue concentrations, indicating efficient clearance from circulation. This pattern supports the notion that silicon acts locally at target sites rather than serving as a circulating reservoir mineral. Unlike calcium or phosphorus, silicon does not appear to be stored in large quantities in a readily mobilizable form, further emphasizing the importance of continuous dietary supply of bioavailable silicon during critical developmental windows [41].

Silicon excretion in poultry is presumed to occur primarily via renal and fecal routes, based on limited avian evidence and analogy with mammalian systems, but definitive characterization of excretion pathways in birds remains lacking [42]. The balance between absorption and excretion is influenced by dietary intake, form, and interactions with other minerals. Kayongo-Male and Julson [38] reported that high dietary silicon levels altered calcium and magnesium metabolism in turkeys, suggesting competitive interactions at absorption or excretion sites. These findings indicate that silicon metabolism is integrated within the broader mineral economy of the bird, rather than operating in isolation.

From a physiological standpoint, silicon’s most consistently documented role in poultry relates to connective tissue metabolism [43]. Early work by Carlisle [4, 5] demonstrated that silicon deficiency results in reduced collagen content in bone and cartilage, leading to structural abnormalities despite adequate mineralization. This observation suggests that silicon is essential for the formation or stabilization of collagen and glycosaminoglycan-protein complexes that constitute the organic scaffold of connective tissues. In poultry, where rapid growth places high demands on skeletal support structures, even subtle impairments in matrix quality can have significant functional consequences.

The involvement of silicon in collagen synthesis has been supported by both biochemical and histological evidence. Carlisle [6] reported that silicon-deficient chicks exhibited markedly reduced collagen concentrations in skull bones, while mineral content remained relatively unaffected. This dissociation between organic and inorganic components underscores silicon’s specific role in matrix formation rather than in mineral deposition. In practical terms, this means that birds may achieve normal body weight and mineral intake yet still suffer from compromised skeletal integrity if silicon availability is inadequate.

Silicon also appears to influence mineral metabolism indirectly by modulating the deposition and retention of other elements within bone tissue. Sgavioli et al. [16] observed increased concentrations of phosphorus, zinc, copper, and manganese in the tibia of broilers receiving silicon supplementation. These trace elements play essential roles in bone enzyme systems and matrix maturation, suggesting that silicon may facilitate a more favorable microenvironment for mineral incorporation. Similarly, Semenenko et al. [44] emphasized silicon’s role as an osteogenic micronutrient that enhances calcium incorporation into bone tissue and supports osteocyte growth and repair.

Beyond skeletal tissues, silicon may exert physiological effects in the gastrointestinal tract that influence overall nutrient utilization. Ruttanavut et al. [39] demonstrated that dietary silicic acid powder increased villus height and epithelial cell proliferation in the duodenum and jejunum of chickens. These morphological changes are indicative of enhanced absorptive capacity and may partly explain improvements in nutrient digestibility reported in studies using ultrafine silicon dioxide. Mustafina and Rakhmatullin [18] reported increased digestibility of dry matter, crude protein, and raw fat in broilers supplemented with ultrafine silicon, suggesting that silicon’s physiological roles extend beyond connective tissue metabolism to encompass digestive function.

At the systemic level, preliminary evidence from a limited number of studies, including conference reports, suggests that silicon supplementation may be associated with considerable variation observed among measured blood biochemical variables reflective of metabolic status; however, these findings should be interpreted cautiously and require confirmation in well-controlled, peer-reviewed studies. Mustafina et al. [45] reported increased total protein and albumin concentrations in the blood of broilers receiving ultrafine silicon, indicating enhanced protein metabolism or improved nutritional status. Increased red blood cell counts and hemoglobin levels were also observed, suggesting potential effects on hematopoiesis or oxygen transport. While these outcomes are not universally reported across studies, they point to broader physiological influences that warrant further investigation.

Importantly, silicon’s physiological roles appear to be most critical during early growth and developmental stages. Carlisle [4] demonstrated that silicon deficiency in young chicks produced severe and rapid effects, whereas older birds exhibited greater tolerance. Scholey et al. [15] similarly reported more pronounced skeletal responses to silicon supplementation in young broilers compared with older birds. This age-dependent sensitivity aligns with the concept that silicon is particularly important during periods of rapid connective tissue synthesis, when demand for matrix components is highest.

Despite these insights, it is equally important to recognize that silicon does not function as a classical metabolic regulator in the same manner as vitamins or hormones. Its effects are subtle, structural, and often permissive rather than overtly stimulatory. This characteristic explains why silicon supplementation may not consistently enhance growth performance or feed efficiency under all conditions, as reported by Elliott and Edwards [14] and Pritchard et al. [20]. Instead, silicon’s physiological value lies in supporting tissue quality, resilience, and long-term functional integrity.

In summary, silicon metabolism in poultry is characterized by selective absorption of soluble forms, rapid tissue uptake, limited systemic storage, and renal excretion. Its physiological roles are centered on connective tissue development, particularly collagen synthesis and bone matrix organization, with secondary effects on mineral metabolism, gut morphology, and overall metabolic status. These functions are most critical during early growth stages and under conditions of high skeletal demand. Appreciating the metabolic and physiological context of silicon provides a foundation for understanding its practical implications for skeletal development and bone health.

## Role of Silicon in Skeletal Development and Bone Mineralization

Skeletal development is a central determinant of productivity, welfare, and longevity in poultry, particularly under modern intensive production systems that demand rapid growth, high feed efficiency, and sustained egg output. The avian skeleton serves not only as a structural framework but also as a dynamic metabolic organ involved in mineral storage, acid-base balance, and hematopoiesis [46, 47]. While calcium, phosphorus, and vitamin D have long been recognized as the primary regulators of bone development and mineralization, growing evidence indicates that silicon plays a distinct and complementary role in skeletal physiology. Unlike classical bone minerals that contribute directly to hydroxyapatite formation, silicon exerts its influence predominantly at earlier stages of bone development by regulating the formation, organization, and maturation of the organic bone matrix [48].

The concept that silicon is essential for skeletal development emerged from carefully controlled deficiency studies conducted in the latter half of the twentieth century. Carlisle [4] provided the first unequivocal evidence that silicon deficiency impairs skeletal growth in chicks. When young cockerels were fed a purified diet low in silicon, they exhibited pronounced growth retardation and skeletal deformities within weeks. Histological examination revealed poorly developed cartilage and bone structures, despite adequate intake of calcium and phosphorus. Importantly, supplementation of the same diet with bioavailable silicon restored normal growth and skeletal architecture, demonstrating a direct causal relationship between silicon availability and bone development.

Subsequent work by Carlisle [5] expanded on these findings by elucidating the mechanistic basis of silicon’s role in skeletal tissues. Using biochemical assays and electron microscopy, Carlisle [5] demonstrated that silicon deficiency resulted in reduced synthesis of collagen and glycosaminoglycans within bone and cartilage. These components constitute the organic matrix that provides tensile strength and serves as a scaffold for mineral deposition. In silicon-deficient chicks, the matrix was poorly organized and structurally weak, leading to abnormal bone morphology even when mineral content appeared relatively normal. This observation was critical in redefining bone health as a function of both organic and inorganic components, with silicon occupying a key position in matrix formation.

One of the most compelling demonstrations of silicon’s skeletal role came from Carlisle [6], who investigated skull formation in chicks under conditions of silicon deficiency. The skull, which undergoes rapid and complex ossification during early development, proved particularly sensitive to silicon availability. Silicon-deficient chicks exhibited severe cranial deformities, including narrowed frontal bones and depressed parietal regions. Biochemical analysis revealed significantly reduced collagen concentrations in skull bones, while calcium and phosphorus levels remained largely unaffected. These findings provided strong evidence that silicon is indispensable for normal bone morphogenesis and that its primary function lies in regulating organic matrix synthesis rather than mineral accretion.

In poultry, skeletal development proceeds through tightly coordinated phases involving chondrogenesis, osteoid formation, mineralization, and remodeling [49, 50]. Silicon appears to be most critical during the early phases of this process, particularly during osteoid formation when collagen fibers and proteoglycans are synthesized and assembled. The presence of silicon in osteogenic cells and their mitochondria, as reported by Carlisle [5], suggests that silicon may influence cellular metabolism at the level of energy production or enzymatic activity required for matrix synthesis. Although the precise molecular mechanisms remain incompletely understood, evidence consistently points to silicon as a facilitator of connective tissue formation.

As poultry genetics have advanced, the relevance of silicon to skeletal health has become increasingly apparent [43]. Modern broilers exhibit growth rates far exceeding those of earlier strains, placing immense mechanical stress on developing bones [51]. This has led to a high prevalence of leg disorders, including tibial dyschondroplasia, valgus varus deformities, and increased susceptibility to fractures [52–54]. While these conditions are multifactorial in origin, compromised bone matrix quality has been identified as a contributing factor. In this context, silicon’s role in enhancing matrix integrity assumes renewed importance.

Experimental studies conducted under practical feeding conditions have provided further insights into silicon’s influence on bone mineralization and strength. Elliott and Edwards [14] evaluated dietary silicon supplementation in broilers and reported no significant effects on body weight gain or tibia ash content. However, closer examination of their findings suggests that while overall mineral deposition was unaffected, subtle changes in bone quality may have gone undetected by conventional ash measurements. This highlights a recurring challenge in silicon research, namely that traditional indicators of bone mineralization may not fully capture improvements in matrix organization and mechanical resilience.

More recent studies employing advanced analytical techniques have yielded clearer evidence of silicon’s skeletal benefits. Sgavioli et al. [16] investigated the effects of soluble silicon supplementation via drinking water on broiler bone composition. Their results demonstrated significant increases in tibial concentrations of phosphorus, zinc, copper, and manganese, elements known to play essential roles in bone enzyme systems and collagen crosslinking. Although tibia ash percentage was not dramatically altered, the observed changes in mineral profile may reflect altered mineral distribution or metabolic activity within bone tissue rather than definitive improvements in bone maturation. These findings may support the notion that silicon facilitates a more efficient and coordinated incorporation of minerals into the developing bone matrix.

The mechanical properties of bone provide a more functionally relevant measure of skeletal health than mineral content alone. Scholey et al. [15] addressed this issue by evaluating tibia breaking strength in broilers supplemented with a novel bioavailable silicon source. Their study demonstrated significant improvements in bone strength and stiffness, particularly in young birds, without corresponding increases in body weight. This decoupling of growth and bone strength underscores silicon’s specific role in enhancing skeletal quality rather than promoting overall growth. Importantly, the improvements in mechanical properties were associated with increased silicon concentrations in bone tissue, providing direct evidence of silicon incorporation into skeletal structures.

Ultrafine and nanoscale silicon sources have further expanded understanding of silicon’s effects on bone development. Mustafina and Rakhmatullin [18] reported that broilers receiving ultrafine silicon dioxide exhibited improved live weight gain and feed efficiency, alongside enhanced mineral utilization. While their study focused primarily on performance parameters, the observed improvements in nutrient digestibility and metabolic efficiency likely contributed to better skeletal support. Nikulin et al. [19] complemented these findings by demonstrating that ultrafine silicon dioxide optimized protein composition in broiler meat, indirectly suggesting improved musculoskeletal development and tissue integration.

Silicon’s role in bone mineralization must also be understood in relation to calcium and phosphorus metabolism. Rather than acting as a direct mineral component of hydroxyapatite, silicon appears to influence the nucleation and orientation of mineral crystals within the collagen matrix. Carlisle [6] hypothesized that silicon may facilitate the initial deposition of calcium phosphate by stabilizing the organic matrix and creating favorable sites for mineral attachment; however, this proposed mechanism requires experimental confirmation in poultry. This hypothesis is supported by observations that silicon deficiency impairs bone formation even when calcium and phosphorus intakes are adequate [6].

Interactions between silicon and other trace minerals further illustrate their integrative role in skeletal metabolism. Zinc, copper, and manganese are essential cofactors for enzymes involved in collagen synthesis and crosslinking [55]. The increased deposition of these elements in bone tissue, observed by Sgavioli et al. [16], suggests that silicon may enhance the functional utilization of trace minerals critical for matrix maturation. Semenenko et al. [44] similarly reported that silicon supplementation improved calcium incorporation into bone tissue and supported osteocyte growth and repair, reinforcing its role as an osteogenic micronutrient.

The developmental timing of silicon availability is a critical determinant of its skeletal effects. Early life stages represent periods of intense bone modeling, during which the organic matrix is laid down at a rapid rate. Carlisle [4] demonstrated that silicon deficiency during these stages produces irreversible skeletal defects, even if silicon is supplied later. Conversely, supplementation during early growth can confer lasting benefits in bone quality. Scholey et al. [15] observed that silicon supplementation was most effective during the starter phase, with diminishing returns as birds aged. This developmental sensitivity underscores the importance of ensuring adequate bioavailable silicon during the earliest stages of poultry production.

In laying hens and breeders, skeletal health has additional implications beyond structural support [56–59]. The avian skeleton serves as a reservoir for calcium mobilized during eggshell formation, particularly from medullary bone [60]. Although the role of silicon in medullary bone metabolism has been less extensively studied, indirect evidence suggests potential benefits. Improved collagen matrix quality may enhance the structural integrity of both cortical and medullary bone, reducing the risk of osteoporosis and fractures in laying hens [61].

It is equally important to consider the limitations and variability of silicon’s skeletal effects. Not all studies have reported changes in measured indicators of bone quality, particularly when insoluble or poorly bioavailable silicon sources are used. Pritchard et al. [20] found that silicon supplementation altered serum mineral profiles in male broilers but did not improve bone density or breaking strength. Such findings highlight the importance of chemical form, dosage, and baseline nutritional status in determining outcomes. Excessive silicon intake may also disrupt mineral balance, as suggested by Kayongo-Male and Julson [38], emphasizing the need for carefully calibrated supplementation strategies.

From a practical perspective, silicon’s contribution to skeletal development should be viewed as supportive rather than substitutive. Silicon cannot compensate for deficiencies of calcium, phosphorus, or vitamin D, nor can it override genetic or management-related factors that predispose birds to skeletal disorders [62]. However, by enhancing matrix quality and facilitating coordinated mineralization, silicon can improve the resilience and functional capacity of the avian skeleton [8, 17, 63, 64]. This role is particularly relevant under conditions of rapid growth, high stocking density, and mechanical stress, where even marginal improvements in bone quality can translate into meaningful gains in welfare and productivity.

In summary, silicon plays a fundamental yet often underappreciated role in skeletal development and bone mineralization in poultry. Its primary function lies in regulating the formation and organization of the organic bone matrix, particularly collagen and glycosaminoglycans, which provide the structural foundation for mineral deposition. In addition to its proposed role in collagen synthesis and extracellular matrix organization during early bone formation, in vitro studies using mammalian osteoblast and mesenchymal cell models have demonstrated that bioavailable silicon can enhance osteoblast proliferation and promote osteoblastic differentiation, potentially through modulation of osteogenic signaling pathways and matrix-related gene expression. Although direct evidence for these cellular mechanisms in avian species is currently limited or unavailable, such findings suggest that silicon may influence bone development not only through structural matrix effects but also via regulation of osteoblast activity. Confirmation of these mechanisms in poultry requires targeted cellular and molecular studies. However, experimental evidence spanning several decades demonstrates that silicon deficiency impairs bone morphogenesis, while supplementation with bioavailable forms enhances bone strength, mineral composition, and overall skeletal integrity. These effects are most pronounced during early developmental stages and are influenced by chemical form, bioavailability, and interactions with other minerals. Recognizing silicon as an osteogenic micronutrient provides a more comprehensive understanding of bone biology in poultry and sets the stage for evaluating its broader impacts on growth performance, nutrient utilization, and production efficiency.

## Effects of Dietary Silicon on Growth Performance, Feed Efficiency, and Carcass Traits

Growth performance, feed efficiency, and carcass quality are core indicators of productivity in poultry production systems [65–67]. Nutritional strategies are traditionally evaluated based on their ability to enhance body weight gain, optimize feed conversion ratio, and improve economically valuable carcass traits while maintaining bird health and welfare [68, 69]. In this context, silicon has historically received limited attention because it does not function as a classical growth-promoting nutrient in the same manner as energy, protein, amino acids, or certain trace minerals [70]. However, an expanding body of research indicates that dietary silicon can indirectly influence growth performance and carcass characteristics through its effects on skeletal development, nutrient utilization, gut morphology, and metabolic efficiency. Understanding these indirect pathways is essential for appreciating silicon’s true nutritional value in poultry.

Early investigations into silicon supplementation and growth performance yielded inconsistent and sometimes contradictory results, contributing to skepticism regarding its practical relevance. Elliott and Edwards [14] evaluated dietary silicon supplementation in broiler chickens and reported no significant effects on body weight gain, feed intake, or feed conversion ratio. These findings reinforced the prevailing view that silicon was nutritionally inert under commercial feeding conditions. However, these early studies often relied on insoluble silicon sources, such as silica or sand, and focused primarily on short-term growth metrics without assessing skeletal quality or physiological adaptations. As later research revealed, such experimental designs were poorly suited to capture silicon’s subtle but biologically meaningful effects.

The relationship between silicon and growth performance is fundamentally indirect. Silicon does not act as an anabolic agent that directly stimulates muscle accretion [10]. Instead, its influence on growth is mediated through improvements in skeletal integrity, connective tissue development, and nutrient absorption. Carlisle [4] demonstrated that silicon-deficient chicks exhibited severe growth retardation, indicating that a minimal level of silicon is essential for normal growth. Importantly, growth impairment in these birds was accompanied by skeletal abnormalities, suggesting that restricted growth was a secondary consequence of compromised structural support rather than impaired energy or protein metabolism per se. This foundational observation established that silicon is permissive for growth, enabling the physical framework necessary for normal body weight gain.

As poultry genetics advanced and growth rates increased, the indirect effects of silicon on performance became more apparent. Modern broilers are selected for rapid muscle deposition, placing extraordinary demands on their skeletal systems [52–54]. When bone development fails to keep pace with muscle growth, birds may exhibit reduced mobility, altered feeding behavior, and increased energy expenditure for postural support [12, 71, 72]. These factors can negatively affect feed efficiency and growth performance. By enhancing the quality and strength of the bone matrix, silicon may alleviate structural limitations that would otherwise constrain growth potential.

More recent studies employing bioavailable silicon sources have reported potential improvements in growth performance and feed efficiency; however, in some cases, these observations are based on limited or preliminary evidence and should be interpreted cautiously. Mustafina and Rakhmatullin [18] investigated the effects of ultrafine silicon dioxide supplementation in broiler diets and observed significant increases in live weight gain compared with control birds. Feed conversion ratio was also improved, indicating more efficient utilization of dietary nutrients. These authors attributed the performance benefits to enhanced digestibility of dry matter, crude protein, and fat, as well as improved metabolic status. Although silicon itself does not supply energy or amino acids, its role in improving nutrient assimilation can translate into tangible performance gains.

Supporting these findings, Nikulin et al. [19] reported that broilers receiving ultrafine silicon dioxide exhibited optimized protein composition in muscle tissue, suggesting improved efficiency of protein utilization and deposition, though this study was preliminary, published in a trade magazine, based on a small sample size, and warrants further confirmation. Enhanced protein quality in meat may reflect improved amino acid absorption, reduced metabolic stress, or more favorable hormonal and enzymatic conditions for muscle growth. However, the conclusions regarding carcass composition or meat quality responses to silicon supplementation remain tentative and should not be generalized without further independent validation. While the precise mechanisms remain to be fully elucidated, these outcomes align with the concept that silicon contributes to a more favorable physiological environment for growth rather than acting as a direct growth stimulant.

The gastrointestinal tract represents a critical interface through which silicon may influence growth performance. Ruttanavut et al. [39] demonstrated that dietary silicic acid powder increased villus height and epithelial cell proliferation in the duodenum and jejunum of chickens. Such morphological adaptations are strongly associated with increased absorptive surface area and enhanced nutrient uptake. Improved gut morphology can reduce the proportion of dietary nutrients lost to endogenous excretion and microbial fermentation, thereby improving feed efficiency. In this way, silicon’s effects on intestinal structure may partially explain observed improvements in growth performance in studies using bioavailable silicon sources.

Sgavioli et al. [16] provided further insight into silicon’s indirect performance effects by supplementing broiler drinking water with soluble silicon. Although overall body weight gain was not significantly altered, the supplemented birds exhibited enhanced tibial mineral content and improved mineral balance. These skeletal improvements may not immediately translate into increased growth rate, but they can support sustained performance by reducing the incidence of leg problems and associated behavioral changes that impair feed intake. In commercial settings, even modest improvements in skeletal robustness can have cumulative effects on flock performance and uniformity.

Feed efficiency, often expressed as feed conversion ratio, is particularly sensitive to factors affecting nutrient utilization and metabolic efficiency. Silicon’s influence on feed efficiency has been reported more consistently than its effects on absolute growth rate. Mustafina et al. [45] observed reduced feed consumption per unit of weight gain in broilers supplemented with ultrafine silicon dioxide, indicating improved feed efficiency. Enhanced digestibility of nutrients, improved gut morphology, and reduced metabolic costs associated with skeletal stress are all plausible contributors to this effect. Birds with stronger skeletal support may expend less energy on locomotion and postural adjustments, allowing a greater proportion of dietary energy to be directed toward growth.

Blood biochemical parameters provide additional evidence linking silicon supplementation to improved metabolic efficiency. Mustafina et al. [45] reported increased total protein and albumin concentrations in the blood of silicon-supplemented broilers, suggesting improved protein metabolism and nutritional status. Higher hemoglobin levels and red blood cell counts were also observed, which may enhance oxygen delivery to tissues and support aerobic metabolism. While these physiological changes do not directly equate to growth promotion, they create favorable conditions for efficient nutrient utilization and tissue accretion.

Carcass traits represent the ultimate expression of growth performance and nutrient partitioning in poultry. Although relatively few studies have explicitly examined the effects of silicon on carcass characteristics, available evidence suggests that silicon may influence meat quality and composition. Nikulin et al. [19] demonstrated that ultrafine silicon dioxide supplementation optimized protein composition in broiler meat, potentially enhancing its nutritional value. Improved protein quality may reflect more efficient muscle fiber development and reduced connective tissue defects, which are influenced by collagen synthesis and crosslinking. Given silicon’s established role in connective tissue metabolism, such effects are biologically plausible. The distribution of muscle mass and the integrity of connective tissue networks within muscle also influence carcass yield and processing characteristics [73]. Stronger connective tissue frameworks can support more uniform muscle development and reduce the incidence of defects such as muscle myopathies [74].

Another aspect of carcass traits influenced by silicon is bone to muscle ratio. Enhanced skeletal development may increase bone mass relative to muscle, potentially affecting dressing percentage. However, studies such as Scholey et al. [15] indicate that silicon supplementation can improve bone strength without significantly increasing bone mass. This distinction is important, as it suggests that silicon enhances bone quality rather than quantity, minimizing potential negative impacts on carcass yield. Stronger bones with optimized matrix composition can support greater muscle loads without excessive increases in skeletal weight.

It is important to recognize that the effects of silicon on growth performance and carcass traits are highly dependent on chemical form, dosage, and baseline nutritional conditions. Studies using insoluble silicon sources have generally failed to demonstrate performance benefits, reinforcing the importance of bioavailability. Elliott and Edwards [14] did not observe growth or feed efficiency improvements with traditional silicon sources, likely due to poor absorption and utilization. In contrast, studies employing soluble, stabilized, or ultrafine silicon forms have reported more consistent positive outcomes.

Baseline diet composition also modulates silicon’s effects. In diets already optimized for energy, protein, amino acids, and minerals, the marginal benefits of silicon supplementation may be modest. However, under conditions of high growth pressure, marginal mineral supply, or suboptimal gut health, silicon’s supportive roles may become more pronounced. Pritchard et al. [20] reported that silicon supplementation altered serum mineral profiles without improving growth performance, highlighting that physiological responses do not always translate into measurable performance gains. Such findings underscore the importance of aligning supplementation strategies with specific production challenges.

Age and production phase further influence silicon’s impact on growth and carcass traits. The greatest responses to silicon supplementation are generally observed during early growth stages, when skeletal development is most rapid and gut morphology is still maturing. Carlisle [4] demonstrated that silicon deficiency during early life produced severe growth impairment, whereas later supplementation could not fully reverse skeletal defects. In agreement, Scholey et al. [15] observed stronger skeletal responses in young broilers. These findings suggest that silicon supplementation strategies aimed at improving growth performance and feed efficiency should prioritize starter and early grower phases.

From a practical standpoint, the economic value of silicon supplementation depends on its cost-effectiveness and consistency of response. While silicon is abundant in nature, bioavailable forms suitable for poultry nutrition may involve additional processing costs. However, improvements in feed efficiency, skeletal robustness, and carcass quality can offset these costs by reducing feed expenses, mortality, and carcass downgrades. Burton et al. [17] emphasized that the adequacy of bioavailable silicon in poultry diets has not been systematically evaluated for decades, suggesting that opportunities exist to refine feeding strategies in light of modern production demands.

In summary, dietary silicon influences growth performance, feed efficiency, and carcass traits in poultry primarily through indirect physiological pathways. By supporting skeletal development, enhancing gut morphology, and improving nutrient utilization, silicon creates a structural and metabolic foundation that enables efficient growth. While silicon does not function as a direct growth promoter, studies using bioavailable forms such as ultrafine silicon dioxide and stabilized silicic acid have demonstrated improvements in weight gain, feed conversion ratio, and meat quality parameters. The magnitude and consistency of these effects depend on chemical form, dosage, developmental stage, and baseline nutritional conditions. Recognizing silicon’s indirect yet meaningful contributions to performance provides a more nuanced understanding of its nutritional value and sets the stage for examining its interactions with minerals, gut health, and nutrient utilization.

## Influence of Silicon on Mineral Interactions, Gut Health, and Nutrient Utilization

The nutritional value of silicon in poultry extends beyond its direct effects on skeletal development and performance outcomes to encompass broader interactions with mineral metabolism, gastrointestinal health, and overall nutrient utilization. These interconnected domains are central to understanding why silicon supplementation can yield physiological and productive benefits even when growth responses are modest or inconsistent. Silicon does not operate in isolation within the avian body. Rather, it functions within a complex network of mineral interactions and digestive processes that collectively determine nutrient efficiency, metabolic balance, and tissue integrity.

### Silicon and Mineral Interactions

Mineral metabolism in poultry is a highly coordinated process involving absorption, transport, storage, and excretion of macro and trace elements. Calcium and phosphorus dominate discussions of mineral nutrition due to their central roles in bone development and eggshell formation. However, trace minerals such as zinc, copper, manganese, magnesium, and iron are equally critical for enzymatic activity, connective tissue synthesis, and antioxidant defense. Silicon interacts with many of these minerals at multiple physiological levels, influencing their bioavailability and functional utilization. Early research by Carlisle [5] highlighted silicon’s close association with calcium metabolism, proposing that silicon facilitates the initial stages of bone mineralization by stabilizing the organic matrix onto which calcium phosphate crystals are deposited. This relationship does not imply that silicon replaces calcium or phosphorus, but rather that it creates a structural and biochemical environment conducive to efficient mineral incorporation. In silicon-deficient chicks, Carlisle [6, 75] observed impaired bone formation despite adequate calcium and phosphorus intake, illustrating that mineral supply alone is insufficient without proper matrix organization.

Empirical evidence supporting silicon’s role in mineral interactions has emerged from studies examining bone mineral composition. Sgavioli et al. [16] demonstrated that broilers receiving soluble silicon supplementation exhibited increased concentrations of phosphorus, zinc, copper, and manganese in the tibia. These trace elements are essential cofactors for enzymes involved in collagen synthesis, crosslinking, and mineral maturation. Zinc is required for alkaline phosphatase activity, copper is critical for lysyl oxidase-mediated collagen crosslinking, and manganese participates in proteoglycan synthesis [76]. The enhanced deposition of these elements suggests that silicon promotes a coordinated mineralization process rather than simply increasing total mineral content. Silicon also interacts with magnesium, an essential cofactor for numerous metabolic enzymes and a regulator of calcium homeostasis. Kayongo-Male and Julson [38] reported that high dietary silicon levels altered calcium and magnesium metabolism in turkeys, indicating potential competitive interactions at absorption or excretion sites. These findings highlight that silicon’s effects on mineral balance are dose-dependent and context-specific. While moderate levels of bioavailable silicon may enhance mineral utilization, excessive intake could disrupt mineral equilibrium, underscoring the importance of precise supplementation strategies. Iron metabolism represents another area of potential interaction. Improved red blood cell counts and hemoglobin levels reported by Mustafina et al. [45] in silicon-supplemented broilers suggest that silicon may indirectly support iron utilization or erythropoiesis. Although the mechanisms remain speculative, enhanced gut health and nutrient absorption could improve iron uptake, while improved connective tissue integrity may support vascular health and oxygen transport efficiency.

### Silicon and Gut Health

The gastrointestinal tract is a critical determinant of nutrient utilization, immune competence, and overall performance in poultry. Increasing evidence indicates that silicon positively influences gut morphology and function, thereby enhancing the efficiency with which nutrients are absorbed and utilized. These effects provide a plausible explanation for improvements in feed efficiency and metabolic status observed in silicon-supplemented birds. Ruttanavut et al. [39] provided direct evidence of silicon’s impact on intestinal morphology by demonstrating increased villus height and epithelial cell proliferation in the small intestine of chickens receiving dietary silicic acid powder. Taller villi increase the absorptive surface area, facilitating greater nutrient uptake per unit of feed consumed. Enhanced epithelial turnover may also improve barrier function, reducing translocation of pathogens and toxins that can compromise nutrient absorption and immune status. The physical properties of certain silicon compounds may further contribute to gut health. Ultrafine silicon dioxide particles, as studied by Mustafina and Rakhmatullin [18], possess high surface area and adsorption capacity, which may enable them to bind harmful substances or modulate microbial populations within the gut. While these effects have not been fully characterized in poultry, analogous mechanisms have been proposed for other inert or semi-inert feed additives, such as clay minerals and aluminosilicates, used to improve gut environment and nutrient efficiency in poultry [77].

Silicon’s role in connective tissue metabolism may also extend to the intestinal mucosa. The gut wall contains a substantial connective tissue component that provides structural support and elasticity. Improved collagen synthesis and matrix organization may enhance intestinal integrity, reducing susceptibility to mechanical damage or inflammation. Although direct evidence in poultry is limited, the consistent association between silicon supplementation and improved gut morphology suggests a meaningful role in maintaining gastrointestinal structure and function. Gut health is closely linked to immune competence, as the intestinal mucosa represents a major interface between the host and the external environment. By supporting epithelial integrity and reducing inflammatory stress, silicon may indirectly enhance immune efficiency, thereby reducing the diversion of nutrients toward immune responses and allowing more efficient allocation toward growth and production. While immune parameters were not the primary focus of most silicon studies, improved metabolic and performance outcomes may partly reflect reduced subclinical immune stress.

### Silicon and Nutrient Utilization

Nutrient utilization encompasses the digestion, absorption, transport, and metabolic use of dietary components, including energy, protein, lipids, vitamins, and minerals. Silicon’s influence on nutrient utilization is multifaceted and reflects its combined effects on gut morphology, mineral interactions, and metabolic efficiency. Protein utilization is a particularly important aspect of poultry nutrition, as protein is both costly and essential for muscle development, enzyme synthesis, and immune function. Mustafina et al. [45] reported increased digestibility of crude protein in broilers receiving ultrafine silicon dioxide, indicating that silicon supplementation can enhance the efficiency with which dietary protein is converted into body protein. Improved protein digestibility may result from enhanced intestinal absorptive capacity, reduced endogenous losses, or improved enzymatic activity.

Nikulin et al. [19] extended these findings by demonstrating optimized protein composition in broiler meat following silicon supplementation. This suggests not only improved protein digestion but also more efficient utilization at the tissue level. Enhanced collagen synthesis and connective tissue organization may support more effective muscle fiber development and protein deposition, contributing to improved meat quality and nutritional value. Energy utilization is another domain influenced by silicon. Improved digestibility of dry matter and fat reported by Mustafina and Rakhmatullin [18] indicates that silicon can enhance the efficiency with which birds extract usable energy from their diets. Improved energy utilization reduces the metabolic cost of maintenance and supports more efficient growth and production. Enhanced oxygen transport capacity, as suggested by increased hemoglobin levels, may further support aerobic metabolism and energy efficiency.

Lipid metabolism may also be indirectly affected by silicon. Improved gut health and nutrient absorption can influence lipid digestion and absorption, while enhanced metabolic efficiency may alter nutrient partitioning between lean tissue and fat. Although direct measurements of lipid metabolism in silicon-supplemented poultry are limited, improved feed efficiency and carcass composition suggest a favorable shift toward lean tissue accretion. Mineral utilization represents a central aspect of silicon’s nutritional value. By enhancing the deposition and functional use of calcium, phosphorus, and trace minerals, silicon improves the efficiency with which these nutrients contribute to skeletal and connective tissue development. Sgavioli et al. [16] demonstrated that silicon supplementation increased tibial concentrations of multiple minerals without increasing total mineral intake, indicating improved utilization rather than increased consumption. Such improvements are particularly valuable in reducing mineral excretion and environmental impact.

### Integration of Effects and Physiological Efficiency

The influence of silicon on mineral interactions, gut health, and nutrient utilization should be viewed as an integrated system rather than as isolated effects. Improved gut morphology enhances nutrient absorption, which in turn supports more efficient mineral utilization and metabolic processes. Enhanced mineral balance supports skeletal integrity and connective tissue function, reducing physiological stress and maintenance energy costs. Together, these effects contribute to improved overall efficiency, even when the growth rate itself is not dramatically altered. This integrative perspective helps explain why silicon supplementation often yields subtle but consistent improvements in feed efficiency, bone quality, and metabolic indicators rather than dramatic increases in body weight. Silicon acts as a functional micronutrient that optimizes biological processes underlying performance rather than directly driving production metrics.

### Limitations and Context Dependence

Despite growing evidence of silicon’s benefits, its effects on mineral interactions and nutrient utilization are context-dependent. Baseline diet composition, mineral balance, silicon form, and inclusion level all influence outcomes. Excessive silicon intake may disrupt mineral equilibrium, as indicated by Kayongo-Male and Julson [38], while poorly bioavailable forms may yield negligible effects. Age and physiological stage further modulate responses, with young birds generally exhibiting greater sensitivity to silicon availability. These considerations highlight the need for precision in silicon supplementation strategies and for further research to define optimal inclusion levels and forms under different production conditions.

In summary, silicon exerts a multifaceted influence on mineral interactions, gut health, and nutrient utilization in poultry. By facilitating coordinated mineral metabolism, enhancing intestinal morphology, and improving the efficiency of protein and energy utilization, silicon supports the physiological foundations of growth, skeletal integrity, and production efficiency. Its effects are indirect yet biologically meaningful, operating through interconnected pathways that optimize nutrient use rather than increasing nutrient supply. Recognizing silicon’s role within this integrated nutritional framework provides critical insight into its value as a functional micronutrient in poultry diets and lays the groundwork for evaluating its specific applications in layers, breeders, and long-term production systems, which will be addressed in the next section.

## Silicon Supplementation in Layers and Breeders: Effects on Egg Quality, Shell Strength, and Reproductive Performance

In laying hens and breeder flocks, nutritional strategies are evaluated not only based on growth or feed efficiency but primarily on their ability to sustain high egg output, maintain egg quality, preserve skeletal integrity, and support long-term reproductive performance. These objectives place unique physiological demands on birds, particularly in relation to mineral metabolism, bone health, and connective tissue function. Silicon, although historically overlooked in layer and breeder nutrition, has emerged as a micronutrient of interest due to its close involvement in bone matrix formation, mineral interactions, and connective tissue integrity. Understanding silicon’s role in these systems is essential for appreciating its potential value in egg production and reproductive management.

### Silicon and Skeletal Integrity

The skeleton of the laying hen serves a dual function: providing structural support and acting as a reservoir for calcium mobilized during eggshell formation [78]. Medullary bone, a specialized bone tissue unique to birds, is rapidly formed and resorbed in response to the daily cycle of egg production [79, 80]. While calcium and phosphorus are the primary constituents of medullary bone, the integrity of the organic matrix is equally critical for maintaining bone strength and preventing fractures [81]. Research on silicon’s role in skeletal development in growing poultry provides a strong foundation for understanding its relevance in layers. Carlisle [5, 6] demonstrated that silicon is essential for collagen synthesis and bone matrix formation. In laying hens, repeated cycles of calcium mobilization place considerable stress on the skeletal matrix, increasing the risk of osteoporosis and fractures, particularly in high-producing strains [82]. Although direct studies on silicon supplementation in laying hens are limited, the established role of silicon in matrix formation suggests that adequate silicon availability may enhance the resilience of both cortical and medullary bone [83].

Lim et al. [84] evaluated a silicate-based complex mineral (SCM) as a dietary additive for laying hens. The inclusion of SCM in the diet increased egg production and feed intake in a dose-dependent manner up to 0.6%, without affecting feed efficiency. Eggshell quality improved, with significantly greater shell thickness and breaking strength compared with the control diet. The authors also observed enhanced bone mineral density at inclusion levels up to 0.4%, together with indicators of improved immunological competence. Overall, the study suggests that silicate-containing mineral complexes can support laying performance, egg quality, and skeletal health in hens, even though the specific metabolic role of silicon was not isolated. However, because such materials exhibit functional properties beyond silicon nutrition, these outcomes may not be attributed unequivocally to silicon acting as a nutrient. Uyanga et al. [37] examined the effects of dietary arginine silicate inositol supplementation on egg quality and bone metabolism in late-phase laying hens. Supplementation at 0.05% improved eggshell strength across the later production phases, while higher inclusion levels (0.10 to 0.15%) increased bone mineral content at 81 weeks of age. Productive performance variables, including body weight, feed intake, feed conversion ratio, and egg output, were unaffected by arginine silicate inositol. However, calcium excretion declined linearly with increasing dietary ASI, indicating enhanced mineral retention, and excretion of several trace minerals also decreased in older hens. Collectively, these findings suggest that arginine silicate inositol can support eggshell integrity and skeletal mineralization in aging layers, likely by improving mineral bioavailability, even though the specific contribution of silicon was not isolated in the study.

Rattanawut et al. [85] evaluated a silicic acid powder containing bamboo vinegar (SPV) as a dietary supplement for laying hens. The authors proposed that silicon may contribute to connective tissue development and digestive function, and the study focused on gut morphology, performance, and egg quality. Supplementation with 2 g/kg SPV increased villus size in the duodenum and jejunum, indicating improved intestinal absorptive capacity. Hens receiving this level of SPV produced more eggs between 34 and 37 weeks of age, and egg mass was higher in birds fed 2–4 g/kg SPV later in the trial. Egg quality traits, including shell and internal characteristics, were not altered. Although not statistically significant, there was a tendency for reduced *Escherichia* coli and *Salmonella* spp. counts in the supplemented groups. These results suggest that silicon in silicic acid-based supplements can enhance gut structure and may support productive performance in laying hens. Yamamoto et al. [86] also explored the influence of dietary silicon supplementation on egg yolk composition and vascular integrity in laying hens. Silicon intake was associated with increases in several free amino acids within the yolk, indicating potential modulation of protein metabolism in the reproductive tract. In addition, the authors reported improved blood vessel strength in supplemented hens, suggesting a supportive role for silicon in connective tissue function. No adverse effects were noted by Yamamoto et al. [86]. Overall, the findings point to potential benefits of silicon for egg composition and vascular health, although its precise nutritional significance in poultry still requires clarification. Furthermore, Semenenko et al. [44] described silicon as an osteogenic micronutrient that enhances calcium incorporation into bone tissue and supports osteocyte growth and repair. These functions are particularly relevant in layers, where continuous bone remodeling occurs throughout the production cycle. Improved matrix quality may reduce microdamage accumulation, maintain bone strength, and lower the incidence of keel bone deformities and fractures, which are major welfare concerns in commercial layer systems [87].

### Silicon and Eggshell Formation

Eggshell quality is a critical determinant of economic efficiency and food safety in egg production. Shell strength, thickness, and integrity are influenced by mineral availability, shell gland function, and the quality of the organic matrix within the shell [88–90]. While calcium carbonate constitutes the bulk of the eggshell, the shell’s mechanical properties depend on a proteinaceous matrix that guides mineral deposition and crystal orientation [91]. Silicon’s role in collagen and glycoprotein synthesis suggests potential involvement in eggshell matrix formation [92]. Although silicon is not a major structural component of the eggshell, its indirect effects on mineral metabolism and matrix organization may influence shell quality. Carlisle [6] proposed that silicon facilitates the initial stages of mineral deposition by stabilizing organic matrices, a mechanism that could plausibly extend to eggshell formation.

Studies examining silicon supplementation in poultry and other avian species have reported improvements in mineral utilization and deposition [92]. Sgavioli et al. [16] demonstrated that silicon supplementation increased tibial concentrations of calcium and trace minerals involved in matrix maturation. Enhanced mineral utilization within bone may reduce competition for calcium between skeletal reserves and eggshell formation, thereby supporting consistent shell quality over time. In breeder hens, where egg quality directly affects hatchability and chick viability, shell integrity is particularly critical. Improved shell strength reduces microbial penetration and moisture loss during incubation, contributing to higher hatch rates [93]. While direct experimental evidence linking silicon supplementation to improved eggshell strength in breeders is limited, the mechanistic basis for such effects is supported by silicon’s established role in connective tissue and mineral metabolism.

### Silicon and Reproductive Performance

Reproductive performance in breeders encompasses egg production rate, fertility, hatchability, and the quality of progeny [94]. These outcomes are influenced by nutritional status, metabolic efficiency, and the integrity of reproductive tissues. Silicon’s involvement in connective tissue formation suggests potential effects on reproductive tract structure and function [62]. The oviduct and associated reproductive tissues contain substantial connective tissue components that provide structural support and elasticity [95]. Adequate collagen synthesis and matrix organization are essential for normal egg formation, transport, and oviposition [96]. By supporting connective tissue metabolism, silicon may contribute to the functional integrity of the reproductive tract, reducing the risk of prolapse, inflammation, or mechanical stress. Blood biochemical indicators reported in silicon-supplemented broilers, such as increased total protein and albumin levels [45], suggest improved overall metabolic and nutritional status. In breeders, such systemic improvements may support reproductive efficiency by ensuring adequate nutrient supply to developing follicles and embryos. Enhanced oxygen transport capacity, indicated by increased hemoglobin levels, may further support embryonic development and viability.

### Silicon and Egg Quality Parameters

Egg quality encompasses a range of parameters, including shell strength, shell thickness, albumen height, yolk color, and internal egg composition. While silicon’s effects on internal egg quality have not been extensively studied, its influence on protein metabolism and mineral interactions suggests potential benefits. Nikulin et al. [19] reported optimized protein composition in broiler meat following silicon supplementation, indicating improved protein utilization at the tissue level. By extension, improved protein metabolism in layers may influence albumen quality, which is primarily composed of proteins synthesized in the oviduct [97]. Enhanced protein synthesis and reduced metabolic stress may support higher albumen height and improved Haugh unit values, key indicators of egg quality [98]. Trace minerals such as zinc and copper, whose deposition in bone tissue was increased by silicon supplementation in broilers [16], are also important for egg quality. Zinc plays a role in carbonic anhydrase activity within the shell gland, influencing shell mineralization, while copper is involved in connective tissue crosslinking [99]. By improving the utilization of these minerals, silicon may indirectly support multiple aspects of egg quality.

### Silicon and Long-Term Production Sustainability

One of the most compelling arguments for considering silicon supplementation in layers and breeders lies in its potential contribution to long-term production sustainability. High egg production places cumulative stress on skeletal reserves, often leading to declines in shell quality and increased fracture risk as hens age [100]. Nutritional strategies that enhance skeletal resilience and mineral efficiency can extend productive lifespan and reduce losses associated with culling and mortality [101]. By improving bone matrix quality and supporting efficient mineral utilization, silicon may help maintain skeletal health over extended laying cycles [37]. This is particularly relevant in modern production systems that aim to extend laying periods beyond traditional cycles. Although direct longitudinal studies in layers are limited, extrapolation from skeletal studies in growing poultry supports the plausibility of such benefits.

### Variability and Research Gaps

Despite the strong mechanistic rationale for silicon supplementation in layers and breeders, empirical data remain limited compared with broiler research. Variability in experimental outcomes may reflect differences in silicon form, dosage, baseline mineral nutrition, and production system. Pritchard et al. [20] demonstrated that silicon supplementation altered serum mineral profiles without improving bone density in broilers, underscoring that physiological responses do not always translate into functional outcomes. These gaps highlight the need for targeted research in laying hens and breeders, particularly long-term studies evaluating bone health, eggshell quality, and reproductive performance under commercial conditions. Such research should prioritize bioavailable silicon sources and consider interactions with calcium, phosphorus, vitamin D, and trace minerals.

**Fig. 1:**
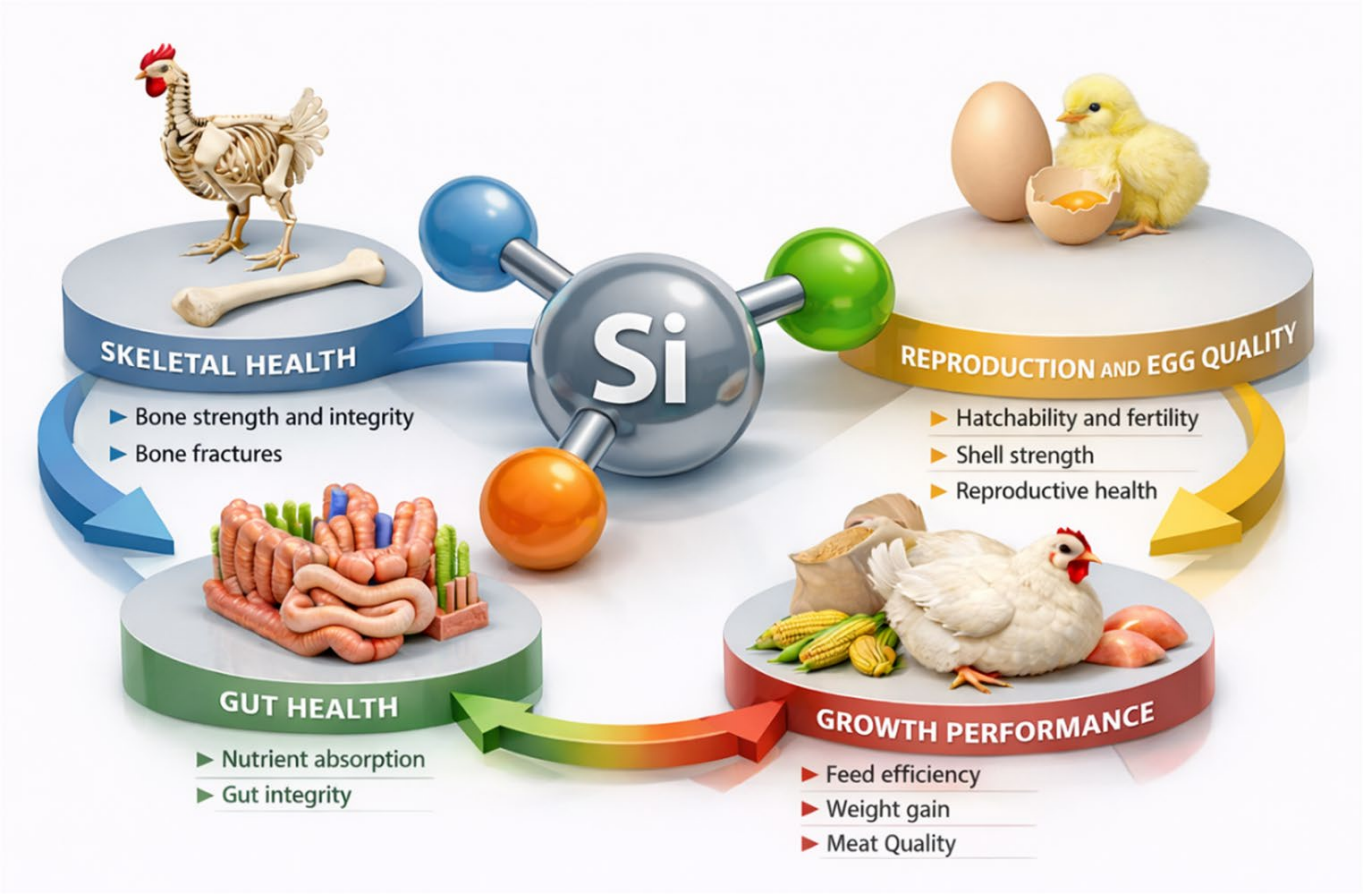
Conceptual illustration of the major biological roles of dietary silicon in poultry, highlighting its effects on skeletal health, gut health, growth performance, and reproduction and egg quality (Figure produced through OpenAI)

In summary, silicon supplementation holds significant potential for supporting egg quality, shell strength, skeletal integrity, and reproductive performance in laying hens and breeders. Through its established roles in connective tissue metabolism, bone matrix formation, and mineral interactions, silicon may enhance the resilience of skeletal and reproductive systems under the demands of high egg production. While direct evidence in layers and breeders remains limited, mechanistic insights and findings from broiler studies provide a strong foundation for further investigation. Recognizing silicon as a functional micronutrient in layer and breeder nutrition opens new avenues for improving welfare, productivity, and sustainability in egg production systems. A summary of key studies evaluating dietary silicon in poultry and reported effects is provided in Table 1. In addition, a conceptual illustration of the major biological roles of dietary silicon in poultry is provided in Fig. 1.

**Table 1:** Summary of key studies evaluating dietary silicon in poultry, including source and form, experimental focus, and reported effects on skeletal health, performance, nutrient utilization, and reproductive traits

Study	Bird type	Silicon source / Form	Main focus	Key impacts reported
Carlisle [4]	Chicks (early growth)	Purified diet, silicon-deficient vs. supplemented	Essentiality, growth, skeletal development	Silicon deficiency caused severe growth retardation, skeletal deformities, and impaired bone development; supplementation restored normal growth
Carlisle [5]	Chicks	Soluble silicon	Connective tissue and bone matrix	Demonstrated silicon is essential for collagen and glycosaminoglycan synthesis in bone and cartilage
Carlisle [6]	Chicks	Silicon-deficient purified diet	Skull formation, bone morphogenesis	Silicon deficiency caused abnormal skull structure and reduced bone collagen despite adequate calcium and vitamin D
Elliott and Edwards [14]	Broilers	Insoluble silicon (silica)	Growth performance, tibia ash	No significant effects on growth or tibia ash, highlighting low bioavailability of insoluble silicon
Carlisle [2]	Review / multiple species	Dietary silicon	Metabolic role of silicon	Identified silicon as a functional trace element involved in early bone formation and connective tissue metabolism
Majewska et al. [33]	Turkeys	Silica grit	Performance and digestion	No beneficial effects; in some cases negative impacts, indicating limited nutritional value of insoluble silica
Ruttanavut et al. [39]	Broilers	Silicic acid powder	Gut morphology	Increased villus height and epithelial cell proliferation in small intestine
Sgavioli et al. [16]	Broilers	Soluble silicon in drinking water	Bone mineral composition	Increased tibial concentrations of P, Zn, Cu, and Mn; improved mineral utilization
Lim et al. [84]	Broilers	Silicon-based supplement	Bone development	Improved tibia mineralization and bone quality indicators
Scholey et al. [15]	Broilers (starter phase)	Stabilized, pH-neutral silicon	Bioavailability, bone strength	Increased silicon bioavailability, improved tibia breaking strength without affecting growth
Burton et al. [17]	Broilers	Novel silica supplement	Bone development	Enhanced bone development and highlighted long-term neglect of silicon adequacy in poultry diets
Pritchard et al. [20]	Broilers (males)	Dietary silicon	Serum minerals, bone traits	Altered serum mineral profiles but no improvement in bone density or strength
Semenenko et al. [44]	Broilers	Dietary silicon	Bone metabolism	Enhanced calcium incorporation into bone and osteocyte growth
Mustafina and Rakhmatullin [18]	Broilers	Ultrafine silicon dioxide	Growth performance, digestibility	Improved weight gain, feed efficiency, nutrient digestibility, and blood biochemical parameters
Mustafina et al. [45]	Broilers	Ultrafine silicon dioxide	Metabolic and hematological traits	Increased serum protein, albumin, hemoglobin, and red blood cell counts
Nikulin et al. [19]	Broilers	Ultrafine silicon dioxide	Meat quality, protein composition	Optimized protein composition of meat and improved nutrient utilization

## Safety, Optimal Inclusion Levels, Limitations, and Future Research Directions

As interest in silicon as a functional micronutrient in poultry nutrition continues to grow, it is essential to evaluate not only its potential benefits but also its safety, appropriate inclusion levels, inherent limitations, and future research needs. Unlike classical nutrients with clearly defined requirements and safety margins, silicon occupies a more complex position due to its abundance in nature, variability in chemical form, and context-dependent biological effects [10]. A balanced assessment of these aspects is critical for translating experimental findings into practical and responsible feeding strategies.

### Safety of Silicon Supplementation

From a toxicological perspective, silicon is generally regarded as safe for poultry when provided in nutritionally relevant forms and amounts. Available evidence suggests that supplementation with bioavailable silicon sources has not been associated with overt adverse effects in poultry at the inclusion levels evaluated in published studies. These reported supplementation levels typically range from tens to several hundred milligrams of added silicon per kilogram of diet or equivalent intake via drinking water, depending on the source and study design. However, total dietary silicon content is rarely quantified, and systematic dose–response studies establishing upper safety thresholds in poultry are limited. Consequently, current assessments of silicon safety in poultry should be interpreted in the context of additive dosage rather than background silicon derived from basal feed ingredients, and further controlled studies are required to define safe upper inclusion levels across different silicon forms. Silicon is ubiquitous in the environment and is naturally consumed by birds through plant-based feed ingredients and water [70]. Available evidence suggests that silicon does not readily accumulate to toxic levels in poultry; however, this conclusion is based on limited data and has not been systematically evaluated across a wide range of supplementation levels or silicon forms. As discussed in the following section, long-term safety data remain limited, particularly for nanoscale silicon forms, and further research is required to establish safe upper inclusion levels under commercial poultry production conditions. Carlisle [2] emphasized that silicon is efficiently regulated through absorption and renal excretion, reducing the risk of systemic accumulation.

Most experimental studies involving silicon supplementation in poultry have reported no adverse effects on health, growth, or survival. Sgavioli et al. [16] supplemented broiler drinking water with soluble silicon and observed no negative effects on performance or mortality. Similarly, Scholey et al. [15] reported improved bone strength without detrimental effects on growth or organ health when broilers were fed a bioavailable silicon supplement. These findings suggest that silicon, when supplied in appropriate forms, is well tolerated by poultry. Semenenko et al. [44] investigated biochemical indicators of bone metabolism in broiler chickens fed diets supplemented with bentonite, a silicon-containing mineral. Silicon was considered an osteogenic micronutrient supporting osteocyte function and bone matrix development. Throughout the trial, survival was 100% in all groups, but birds receiving bentonite showed better general condition, including higher activity and healthier feathering. Growth performance improved, with average daily gain exceeding that of the control group during all monitored stages. In contrast, control birds displayed reduced mobility and lower weight gains toward the end of the growing period. Bentonite supplementation also altered several blood biochemical markers related to mineral metabolism, suggesting more favorable conditions for bone calcification and structural integrity. However, these effects may reflect the functional properties of the additive rather than a direct nutritional role of silicon per se.

However, safety considerations are closely linked to the chemical form of silicon. Insoluble crystalline silica, while largely inert in the gastrointestinal tract, poses occupational health risks when inhaled as dust but is not associated with dietary toxicity in poultry [9, 10, 70, 102]. In contrast, ultrafine or nanoscale silicon dioxide, although shown to enhance bioavailability and performance outcomes, warrants careful evaluation due to its high reactivity and surface area. Mustafina and Rakhmatullin [18] reported positive effects of ultrafine silicon dioxide on broiler performance without adverse outcomes, but long-term safety data remain limited. This highlights the importance of distinguishing between dietary safety and handling safety, as well as between short-term and lifetime exposure. Excessive silicon intake may disrupt mineral balance rather than induce direct toxicity. Kayongo-Male and Julson [38] demonstrated that high dietary silicon levels altered calcium and magnesium metabolism in turkeys, suggesting potential antagonistic interactions at elevated inclusion rates. These observations underscore that silicon effects are dose-dependent and context-specific, and that indiscriminate supplementation, particularly at high inclusion levels, may adversely affect calcium and magnesium utilization rather than confer universal benefits. While such effects were not associated with overt toxicity, they underscore the need for moderation and precision in supplementation.

### Optimal Inclusion Levels and Practical Considerations

One of the central challenges in silicon nutrition is the absence of clearly defined dietary requirements or recommended inclusion levels for poultry. Unlike calcium or phosphorus, silicon requirements have not been established in nutrient requirement tables, largely because overt deficiency is unlikely under practical feeding conditions. However, the distinction between total dietary silicon and bioavailable silicon complicates this issue. Studies employing purified diets have demonstrated that extremely low silicon intake results in severe deficiency symptoms, as shown by Carlisle [4]. In contrast, conventional poultry diets often contain high total silicon concentrations derived from plant materials and soil contamination. The key question, therefore, is not whether diets contain silicon, but whether they provide sufficient bioavailable silicon to support optimal physiological function.

Pritchard and Nielsen [43] conducted an umbrella review synthesizing evidence on silicon supplementation and skeletal health across animal and human studies. Although this work did not focus on poultry, it reported generally positive associations between silicon intake and bone and mineral metabolism across species. The authors proposed an approximate intake threshold of 139 mg silicon/kg body weight per day for measurable skeletal effects in animals; however, they emphasized substantial variability in study design, dosage, and silicon bioavailability, and noted that such intake levels may not be physiologically achievable or relevant for many species. Importantly, due to marked species differences in metabolism and skeletal physiology, these findings should be interpreted as supportive rather than directly transferable to poultry. The review, therefore, underscores the need for poultry-specific, controlled studies to determine biologically relevant intake ranges, safety margins, and practical applicability.

Experimental studies provide some guidance on effective supplementation levels, although comparisons are complicated by differences in silicon form and delivery method. Sgavioli et al. (2016) reported beneficial effects on bone mineral composition using silicon supplied through drinking water at relatively low concentrations. Scholey et al. [15] demonstrated improved bone strength using a stabilized silicon supplement at inclusion levels that did not alter feed intake or growth. Mustafina and Rakhmatullin [18] observed performance benefits with ultrafine silicon dioxide at low dietary inclusion rates, emphasizing that bioavailability rather than quantity is the primary determinant of efficacy. From a practical standpoint, silicon supplementation should aim to provide modest amounts of highly bioavailable silicon, particularly during early growth phases or periods of high skeletal demand. Excessive supplementation is unlikely to yield additional benefits and may increase the risk of mineral imbalances. Water-based supplementation offers an alternative approach that allows precise control of intake and may be particularly effective during critical developmental windows.

### Limitations of Current Knowledge

Despite significant advances, several limitations constrain the application of silicon nutrition in poultry. One major limitation is the variability of experimental outcomes. While some studies report improvements in bone strength, feed efficiency, or nutrient utilization, others observe minimal or no effects. Pritchard et al. [20] demonstrated alterations in serum mineral profiles without corresponding improvements in bone density or performance, highlighting the complexity of silicon’s biological role. Another limitation is the lack of standardized analytical methods for assessing bioavailable silicon in feeds and biological tissues. Total silicon content provides limited insight into nutritional value, and differences in analytical techniques complicate comparisons across studies. Improved methods for quantifying soluble and metabolically active silicon would greatly enhance research consistency and practical application.

The interaction of silicon with other dietary components represents an additional challenge. Silicon’s effects are influenced by calcium, phosphorus, magnesium, and trace mineral status, as well as by vitamin D and overall diet composition. Without careful control of these variables, it is difficult to isolate silicon’s specific contributions or to predict responses under commercial conditions. Long-term studies in laying hens and breeders are particularly scarce. While mechanistic evidence supports potential benefits for bone health and egg quality, empirical data under extended production cycles are limited. This gap restricts the ability to make definitive recommendations for these production systems.

### Future Research Directions

Future research on silicon in poultry nutrition should prioritize several key areas. First, there is a need to establish functional benchmarks for bioavailable silicon intake under different production scenarios. Rather than defining a single requirement, research should aim to identify optimal ranges that support skeletal integrity, nutrient utilization, and production efficiency without disrupting mineral balance. Second, comparative studies evaluating different silicon sources are essential. Research should systematically compare insoluble, soluble, stabilized, organic, and ultrafine silicon forms under standardized conditions to clarify their relative bioavailability, efficacy, and safety. Such studies would provide practical guidance for feed formulation and supplementation strategies.

Third, mechanistic research at the molecular and cellular levels is needed to elucidate how silicon influences collagen synthesis, mineral nucleation, and gut morphology in poultry. Advances in molecular biology and imaging techniques offer opportunities to clarify silicon’s role in osteogenic and epithelial cells, bridging the gap between observed outcomes and underlying processes. Fourth, long-term and life-cycle studies in layers and breeders are critical. These studies should evaluate the effects of silicon supplementation on bone health, eggshell quality, reproductive performance, and welfare over extended production periods. Such data are essential for assessing silicon’s value in sustainable egg production systems. Finally, interactions between silicon and gut microbiota represent an emerging area of interest. Given silicon’s influence on gut morphology and nutrient utilization, its potential effects on microbial composition and function warrant investigation. Understanding these interactions could reveal additional pathways through which silicon supports poultry health and performance.

### Concluding Perspective

In summary, silicon represents a safe and biologically meaningful micronutrient with the potential to enhance skeletal integrity, nutrient utilization, and production sustainability in poultry. While it is not a classical growth-promoting nutrient, its supportive role in connective tissue metabolism, mineral interactions, and gut health positions it as a valuable functional component of modern poultry diets. The absence of defined requirements and variability of responses reflects gaps in knowledge rather than a lack of biological relevance. With targeted research and careful application, silicon supplementation has the potential to contribute meaningfully to poultry nutrition, welfare, and productivity in the years ahead.

## Overall Conclusions

Silicon has traditionally been seen as nutritionally insignificant in poultry diets because of its abundance in plant-based feed ingredients and the lack of obvious deficiency symptoms under commercial conditions. However, evidence compiled in this review clearly shows that silicon is a biologically important micronutrient with key supportive roles in poultry physiology. Early deficiency studies conducted under purified dietary conditions demonstrated that silicon plays a fundamental biological role in normal growth and skeletal development. Under conditions of limited silicon availability, impaired collagen synthesis, abnormal bone formation, and reduced structural integrity were observed despite adequate calcium and phosphorus intake, highlighting silicon’s role in the organic bone matrix rather than as a direct mineral component. However, the extent to which these deficiency-based findings translate to modern commercial poultry production systems remains uncertain and warrants further investigation using contemporary diets and management conditions. Based on the collective evidence reviewed, silicon can be regarded as biologically essential, as demonstrated by early deficiency studies conducted under purified dietary conditions, where inadequate silicon intake resulted in impaired growth, abnormal skeletal development, and defective collagen synthesis despite sufficient calcium and phosphorus supply. However, under practical commercial poultry nutrition conditions, silicon cannot currently be classified as an essential nutrient, as conventional plant-based diets provide substantial background silicon and overt deficiency symptoms are unlikely to occur. Consequently, silicon is best described as non-essential in practical terms but biologically important, with potential conditionally beneficial effects depending on dietary composition, silicon form, bioavailability, and physiological or production stressors. Further controlled studies using commercial-type diets are required to determine whether silicon should be considered conditionally essential under specific production scenarios.

A consistent conclusion across studies is that the nutritional value of silicon depends mainly on its chemical form and bioavailability, not on the total dietary amount. While standard diets contain significant silicon, much of it is poorly soluble and biologically inactive. Conversely, soluble, stabilized, organic, and ultrafine silicon sources show greater absorption and physiological benefits. These forms have been linked to improvements in bone strength, mineral utilization, gut health, and overall metabolic efficiency.

In broiler production, silicon indirectly supports growth performance and feed efficiency by promoting skeletal health, connective tissue quality, and nutrient absorption. Although silicon does not function as a traditional growth promoter, its role in boosting feed efficiency, protein use, and carcass quality becomes increasingly important under the high growth demands of modern poultry breeds. The same principles apply to laying hens and breeders, where silicon’s role in bone matrix formation and mineral interactions suggests potential benefits for eggshell quality, skeletal health, and long-term reproductive success, even though targeted studies in these areas are limited.

Overall, silicon should be viewed as a safe, functional micronutrient that enhances structural and metabolic processes, rather than merely increasing nutrient supply. Future research should focus on identifying effective inclusion levels, comparing bioavailable silicon sources, and assessing long-term effects in layers and breeders. Recognizing silicon’s integrated role provides a more comprehensive understanding of poultry nutrition and supports its consideration in strategies to improve productivity, welfare, and sustainability.

## Data Availability

No datasets were generated or analysed during the current study.
